# Land use imperils plant and animal community stability through changes in asynchrony rather than diversity

**DOI:** 10.1038/ncomms10697

**Published:** 2016-02-12

**Authors:** Nico Blüthgen, Nadja K. Simons, Kirsten Jung, Daniel Prati, Swen C. Renner, Steffen Boch, Markus Fischer, Norbert Hölzel, Valentin H. Klaus, Till Kleinebecker, Marco Tschapka, Wolfgang W. Weisser, Martin M. Gossner

**Affiliations:** 1 Department of Biology, Technische Universität Darmstadt, Schnittspahnstrasse 3, D-64287 Darmstadt, Germany; 2 Terrestrial Ecology Research Group, Department for Ecology and Ecosystem Management, Center for Life and Food Sciences Weihenstephan, Technische Universität München, Hans-Carl-von-Carlowitz-Platz 2, D-85354 Freising, Germany; 3 Evolutionary Ecology and Conservation Genomics, University of Ulm, Albert-Einstein-Allee 11, D-89069 Ulm, Germany; 4 Institute of Plant Sciences, University of Bern, Altenbergrain 21, CH 3013 Bern, Switzerland; 5 Institute of Zoology, University of Natural Resources and Life Sciences, Gregor-Mendel-Strasse 33, 1180 Vienna, Austria; 6 Smithsonian Conservation Biology Institute at the National Zoological Park, Front Royal 22630, Virginia, USA; 7 Senckenberg Gesellschaft für Naturforschung, Biodiversity and Climate Research Centre (BiK-F), D-60325 Frankfurt, Germany; 8 Institute of Landscape Ecology, University of Münster, Heisenbergstrasse 2, D-48149 Münster, Germany

## Abstract

Human land use may detrimentally affect biodiversity, yet long-term stability of species communities is vital for maintaining ecosystem functioning. Community stability can be achieved by higher species diversity (portfolio effect), higher asynchrony across species (insurance hypothesis) and higher abundance of populations. However, the relative importance of these stabilizing pathways and whether they interact with land use in real-world ecosystems is unknown. We monitored inter-annual fluctuations of 2,671 plant, arthropod, bird and bat species in 300 sites from three regions. Arthropods show 2.0-fold and birds 3.7-fold higher community fluctuations in grasslands than in forests, suggesting a negative impact of forest conversion. Land-use intensity in forests has a negative net impact on stability of bats and in grasslands on birds. Our findings demonstrate that asynchrony across species—much more than species diversity alone—is the main driver of variation in stability across sites and requires more attention in sustainable management.

## Abstract (web summary)

Long-term stability of ecological communities is vital for maintaining ecosystem functioning. Here, Blüthgen *et al*. show that greater land-use intensity in grasslands and forests can have negative impacts on the stability of plant and animal communities, driven primarily by variation in asynchrony between species.

## Graphical abstract

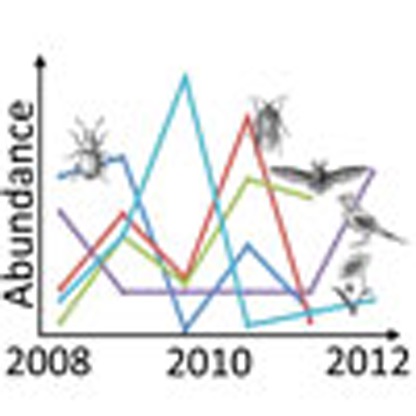

The long-term functional stability of ecosystems is driven by the stability of species' populations and communities that contribute to ecosystem functions. Community stability thus represents a main goal for biodiversity conservation and sustainable management of natural resources^1^,^2^,^3^. Populations of single species tend to fluctuate over time, an instability that is commonly quantified by the coefficient of variation in abundance across years: CV=*σ*/*μ*, where *σ* is the s.d. of the species' abundance over time and *μ* its temporal mean. Stability is the inverse of the coefficient of variation (CV^−1^)^4^. Unless species abundances are perfectly synchronous, the total community CV_tot_ must be smaller than the average species' CV_sp_. The reduction in CV_tot_ relative to CV_sp_ increases with the number of species as well as with their asynchrony^4^,^5^. This stabilization is known as portfolio effect^4^ and insurance hypothesis^6^, respectively, in analogy to financial market theory and is comparable to risk minimization in financial investments. The portfolio metaphor emphasizes the statistical averaging effect of multiple species, while the insurance hypothesis explicitly describes the species' asynchrony in environmental responses and their dynamics^7^. Asynchrony has been recognized as a key driver behind a positive diversity–stability relationship^8^,^9^. In principle, asynchrony and its positive contribution to stability can mirror interspecific competition, heterogeneity in species responses to environmental conditions (response diversity) or simply demographic stochasticity^5^,^10^,^11^,^12^,^13^. Total abundance may additionally influence stability in several ways either indirectly via changes in the variance (*σ*) or directly as its mean is the denominator in the ratio (*σ*/*μ*) that defines CV_tot_ (refs 8, 14).

Empirical support for the diversity–stability relationship and its underlying drivers in terrestrial ecosystems mainly comes from primary producers, such as plants in experimentally or naturally assembled grasslands^4^,^11^,^14^,^15^,^16^,^17^,^18^ and trees in mixed forests^19^. A few studies examined the effect of fertilization on inter-annual asynchrony and stability of grassland plant communities, with conflicting results^11^,^17^,^18^. It remains unknown whether findings on grassland plants can be generalized for other taxa and trophic levels, for other ecosystems such as forests or for other land-use impacts. Given that land-use change is a main driver of biodiversity loss^20^, conversions between habitat types and intensification of land use within habitat types should also affect community stability.

Our study evaluates the changes in stability of plant and animal communities across 4–6 years in naturally assembled forests and grasslands under characteristic land-use regimes in three regions in Germany (50 forests and 50 grasslands per region). Analyses were performed for four taxonomic groups, encompassing a total of 562 plant, 1,982 arthropod, 114 bird and 13 bat species. We chose sampling methods that adequately represent the whole community within a taxon.

We hypothesized that changes in land-use type and increasing land-use intensity have negative net effects on community stability via reduced diversity, asynchrony and abundance. Habitat conversion from (managed) forest to cultivated grassland and arable land has been the main change in land uses in vast parts of the Central European cultural landscape for many centuries, although this trend is now partly reversed^21^. Therefore, we first tested whether forests have more stable communities than grasslands, reflecting the historical deforestation and conversion. Secondly, we evaluated whether gradual increases in land-use intensity within forests and grasslands additionally destabilize communities. Finally, we modelled the indirect effects of land-use intensity on stability via changes in diversity, asynchrony and total abundance using structural equation modelling. Our study revealed that arthropod and bird communities were more stable in forests than in grasslands. Land-use intensity had negative impacts on the stability of bats in forests and birds in grasslands. In contrast, plant communities were not destabilized through increased land-use intensity, despite strong diversity declines in highly fertilized and frequently mown grasslands. Overall, stability was most strongly driven by asynchrony, followed by diversity and abundance, confirming a key role of the insurance effect in real-world ecosystems.

## Results and Discussion

### Variation in community stability

**Figure 1:**
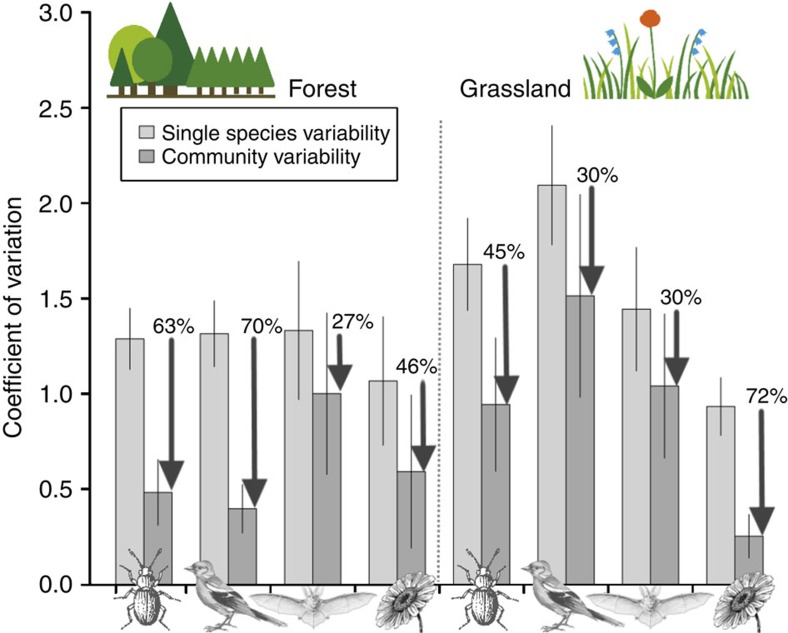
Stabilization gain by communities compared with single species. Communities had a lower inter-annual variability (coefficient of variation, CV_tot_) in total abundance than single species (CV_sp_). The figure shows strong decreases in CV_tot_—and thus increased stability (arrows)—compared with the mean CV_sp_, resulting from portfolio effects and species asynchrony. Four taxa (arthropods, birds, bats and plants) in forests and grasslands were compared. Differences in stability between forests and grasslands in interaction with taxon were highly significant, whereas the relative stability gain (CV_sp_/CV_tot_) between the two habitats was not. Each bar shows mean±s.d. across all plots (*N*=135–150 plots, except forest arthropods: *N*=30).

Inter-annual variability of the total abundance of all species in a community (CV_tot_) was much lower than the mean species-level variability (CV_sp_) in both forests and grasslands (Fig. 1). This stabilizing effect ranged from a 27% lower CV_tot_ of forest bats to a 70% lower CV_tot_ in resident forest birds and even a 72% lower CV_tot_ in cover of grassland plants compared with the respective mean CV_sp_ (arrows in Fig. 1). Community stability (CV_tot_^−1^) was significantly reduced in grasslands compared with forests, but the effect differed across taxa (Supplementary Tables 1 and 2). For arthropods and birds, we found a 2.0- and 3.7-fold decrease, respectively, in community stability from forests to grasslands, whereas no differences were observed in bats. This destabilization was associated with lower asynchrony and higher level of CV_sp_ in grassland arthropods and birds (Supplementary Table 2). Plants had the most stable communities and showed the opposite trend, that is, grassland communities and populations were more stable and asynchronous than the forest understory vegetation. Note, however, that we excluded the tree and shrub layers, hence the most stable vegetation layers, from the analysis in forests.

**Figure 2:**
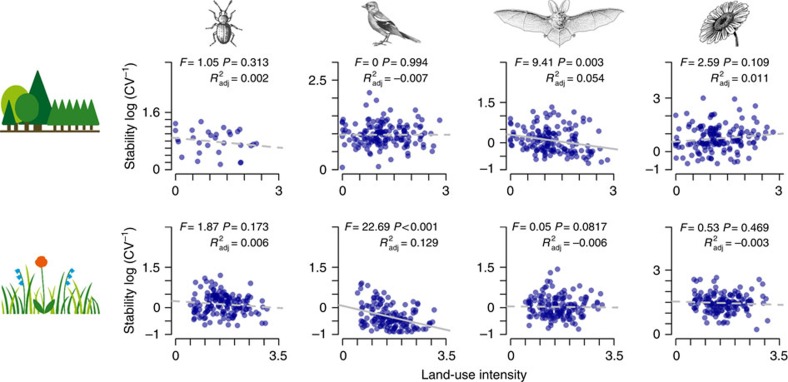
Effect of land-use intensity on community stability within forests and grasslands. Changes in community stability (calculated as the inverse of community variability, CV_tot_^−1^) with the combined land-use intensity in forests (top row) and grasslands (bottom row) are shown for each of the four taxa. Significance values are derived from linear models.

Land-use intensity gradients within forests and within grasslands showed relatively weak effects on community stability, which were only significant for grassland birds and forest bats (Fig. 2). Land-use impacts on animal community stability were thus stronger for conversion of forests into open grassland than for gradual intensity variation, partly corresponding to a study on tropical birds^22^. Moreover, results for grassland plants—the taxon that has received most attention so far—may not fully represent the potential land-use responses of other organisms. Stability of arthropods, birds and bats negatively responded to conversion and/or intensity, whereas plants did not, supporting the idea that higher trophic levels can show increased sensitivity to land use and accelerate the losses of plant diversity^23^,^24^. Stability theories for consumers often focused on the roles of food-web structure, species mobility or body size^25^,^26^. While such food-web approaches often developed separately from merely plant-centred views^27^, insights from empirical studies across trophic levels—based on a common methodology and stability concept as in our study—may stimulate unified theories of stability in the future.

### Drivers of stability

Generally, high instability (CV_tot_=*σ*/*μ*) may arise from high standard deviation (*σ*) or from a low mean of the total abundance (*μ*), suggesting that either the community density or fluctuations are critical. In all communities in our study, *σ* was consistently more variable across sites than *μ* (Supplementary Table 2), suggesting an important role of variance-driven instability. The negative land-use intensity effect on stability for forest bats (Fig. 2) was driven by a significant decline in *μ*, which was not compensated by a decline in *σ*. The negative land-use effects on stability for grassland birds were, however, neither accompanied by significant changes in *μ* nor in *σ* (Supplementary Figs 5 and 6).

**Figure 3:**
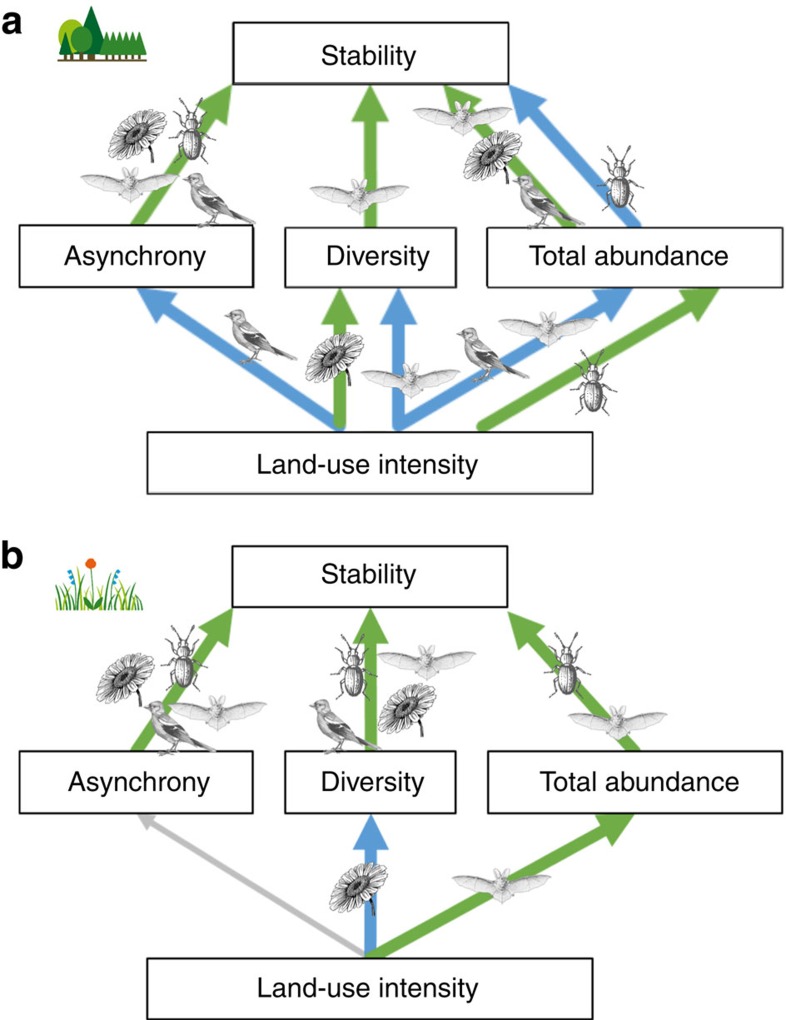
Indirect effects of land-use intensity on community stability. Results from structural equation models are summarized over all taxa for forests (**a**) and grasslands (**b**). Community stability (the inverse of variability) is affected via different stabilizing and destabilizing paths. Green arrows show positive, blue negative directional effects. Taxon symbols next to arrows indicate significant effects (*P*<0.05) for the respective taxon.

Gradual increases in land-use intensity indirectly affected stability through changes in asynchrony, diversity and abundance (Fig. 3). Despite differences in the strength and significance of the three stabilizing pathways between taxa, land-use intensity generally decreased at least one of them (Fig. 3 and Supplementary Figs 2 and 3). The strongest effects occurred via asynchrony and abundance for all taxa in forests, with an additional strong effect via diversity for forest bats. In grasslands, the strongest effects of land-use intensity on stability also occurred via asynchrony for all taxa, and additional effects via diversity were also consistent across all taxa. Land-use effects on animal community stability most likely also translate into changes in their ecosystem functions, for example, consumption-related processes such as parasitism, predation, herbivory, decomposition or pollination^28^. Hence, we also analysed herbivorous and carnivorous arthropods separately and found marked differences in how land-use intensity affects their stability. Forest land-use intensity reduced the stability of carnivores via asynchrony and abundance, whereas herbivore stability was affected via diversity (Supplementary Fig. 4). Although grassland herbivore communities might become more unstable when fewer plant species are available, as shown in experimental grasslands^24^, the negative effects of land-use intensity on plant diversity did not translate into reduced stability of herbivorous arthropods in our study.

Diversity decline in response to land use was not prevalent in all taxa, corresponding to earlier findings in the same sites^29^,^30^,^31^ and elsewhere^32^. Hence, species diversity alone does not sufficiently capture critical changes in communities and ecosystems. In addition, different components of land use can have variable or even contrasting effects (Supplementary Figs 2 and 3). In forests, harvesting of trees had the strongest negative impact on birds and bats, whereas non-natural tree species affected plants and arthropods most strongly. The proportion of non-natural trees also contributed to reduced bird abundance but increased plant and arthropod diversity. In contrast, the amount of dead wood with saw cuts was positively related to bird asynchrony. In grasslands, increases in bat abundance were driven by grazing intensity. Increased mowing and fertilization intensity as well as grazing intensity were associated with a lower diversity of plants, whereas mowing and fertilization increased the plants' asynchrony and abundance. Land-use intensity thus showed a negative impact on diversity, but this was compensated by asynchrony and abundance; thus, no net effect on stability was found. This finding corresponds to a study conducted in a grassland site in Michigan, where fertilization increased the asynchrony of plant communities and compensated for destabilizing diversity losses^11^. In contrast, in a Mongolian semiarid grassland plant community, the addition of nitrogen and phosphorous was found to be destabilizing for the plant community, whereas mowing had a stabilizing effect^17^. In a global meta-analysis, Hautier *et al.*^18^ also showed that plant communities were destabilized when grasslands were fertilized, partly via a decreased asynchrony.

Asynchrony significantly increased stability in all taxa and had by far the strongest impact on community stability in all taxa, except in forest bats where diversity had an equally strong influence. The primary importance of asynchrony was unaffected when an unweighted synchrony index was used (see Supplementary Tables 3 and 4). Diversity also contributed to stability in all grassland communities. Abundance had a stabilizing effect for birds, bats and plants in forests and for bats and arthropods in grasslands, but was destabilizing for forest arthropods (Fig. 3). Asynchrony, diversity and abundance were often correlated (Supplementary Figs 2 and 3). Diversity was positively correlated with asynchrony in forest bats and grassland arthropods, suggesting that common mechanisms may jointly affect both, or that compensatory dynamics increase with diversity. In contrast, plants, birds and bats in grasslands showed negative correlations between diversity and asynchrony. Different, complementary conservation and management actions in grasslands may thus be required when aiming to stabilize communities via both diversity and asynchrony. Interestingly, the decrease in asynchrony with higher diversity of grassland plants contrasts with the opposite trend reported along plant richness gradients in experimental^9^ and naturally assembled grasslands^18^.

### Outlook

Several studies found that the composition of species and their functional traits, functional performances or interactions can show marked changes with land use even when diversity remains unchanged^29^,^31^,^33^. Some of these functional traits can be relevant for stability, for example, thermal niches^33^ or phenotypic plasticity^34^. Our results strongly support the view that other measures such as traits or inter-annual variability should complement diversity surveys to evaluate potential impacts of global change. We show that, despite the unequivocal positive diversity–stability relationship, diversity alone, without knowledge of the level of asynchrony, may be a poor indicator of community stability. Stabilization of communities and of their functional performance via diversity is an established goal for sustainable ecosystem management^2^. However, so far conservation assessments evaluating land-use impacts or the impact of land sharing versus land sparing between natural habitats and agriculture^35^ are almost exclusively based on short-term diversity surveys. We suggest that future sustainable management concepts should also target and monitor processes and spatial habitat requirements that facilitate compensatory dynamics to promote inter-annual asynchrony as a key factor driving long-term stability.

## Methods

### Study system and definition of land-use intensity

Our study was conducted within the framework of the Biodiversity Exploratories programme, which includes grasslands and forests within three regions in Germany: (1) Schwäbische Alb in South-west Germany (48°34′ to 48°53′N; 9°18′ to 9°60′E), (2) Hainich-Dün in central Germany (50°94′ to 51°38′N; 10°17′ to 10°78′E) and (3) Schorfheide-Chorin in North-east Germany (52°47′ to 53°13′N; 13°23′ to 14°09′E). Within each region, 50 plots of 100 m × 100 m size were chosen within forests and 50 plots of 50 m × 50 m size on managed grasslands^36^. The plots were selected to represent the regional range of management intensity for both forests and grasslands.

The forest plots cover different management strategies (unmanaged beech forests, even-aged age-class forests or uneven-aged selection-cutting forests), different main tree species within managed age-class forests (European beech *Fagus sylvatica*, Norway spruce *Picea abies*, Scots pine *Pinus sylvestris*) and different developmental stages within managed age-class forests (for example, thicket stage, pole wood, young timber and old timber). Unmanaged beech forests were set aside from management 20–70 years ago, but have been managed before. No pristine forests exist in Central Europe. Our land-use intensity measure for forests is based on inventory data of the living stand, stumps and dead wood, and includes the following three components^37^: (1) the proportion of harvested tree volume (*Iharv*), (2) the proportion of trees provided by species that are not part of the natural forest community (*Inonat*) and (3) the proportion of dead wood showing signs of saw cuts (*Idwcut*). The single components were used in structural equation models, while the compound index (*ForMI*) was used for main regressions. The compound *ForMI* index for each plot *i* is the sum of its three components, that is, *ForMI*_*i*_=*Iharv*_*i*_+*Inonat*_*i*_+*Idwcut*_*i*_ ranging from zero (unmanaged forest) to three (intensively managed forest).

In grasslands, land-use intensity includes the three components fertilization, mowing and grazing, often applied in combination (meadows, pastures and mown pastures)^38^. Land use was assessed for each plot yearly from 2006 to 2012 through standardized questionnaires with the land owners. Fertilization intensity *F*_*i*_ was defined as the amount of nitrogen applied (kg nitrogen per ha per year), mowing *M*_*i*_ as the frequency of cuts per year on each site *i* and grazing *G*_*i*_ was quantified as the livestock density (livestock units per days per ha per year). Each land-use component was standardized against its mean across regions (*F*_m_, *M*_m_, *G*_m_) as *F*_std_=*F*_*i*_/*F*_m_, *M*_std_=*M*_*i*_/*M*_m_ and *G*_std_=*G*_*i*_/*G*_m_, and averaged over all years. For regressions, a compound index of land-use intensity^38^ was used as follows: *LUI*_*i*_=*F*_std_+*M*_std_+*G*_std_. The *LUI*_*i*_ was calculated for each year, square-root-transformed for normality and then the mean *LUI*_*i*_ of the 7 years (2006–2012) was used. The *LUI* index has been shown to predict responses in the vegetation, namely the plants' nitrogen indicator values, nitrogen and phosphorous contents in plant and soil as well as plant diversity^38^.

For the structural equation models, mowing and fertilization were combined in a single factor as (*F*_std_+*M*_std_)/2 because they were non-independent, that is, most plots were either fertilized and mown (69 of 150 plots), or unfertilized and not mown (34 plots). This positive coupling of both treatments was highly significant in a *χ*^2^-test of homogeneity (*χ*^2^=29.6, degree of freedom=1, *P*<10^−7^; Supplementary Table 5). In contrast, grazing was independent of mowing and fertilization. There was a significantly negative association with fertilization (*χ*^2^=13.3, *P*=0.0003) and mowing (*χ*^2^=3.9, *P*=0.047), that is, these treatments were more commonly applied alone than in combination (Supplementary Table 5). Moreover, fertilization intensity and mowing frequency were highly positively correlated, whereas grazing intensity was negatively related to these two treatments. The correlation between (sqrt-transformed) fertilization (✓*F*_std_) and mowing intensity (*M*_std_) was highly significantly positive (*r*=0.65, *P*<10^−15^), whereas grazing (✓*G*_std_) was negatively related to ✓*F*_std_ (*r*=–0.30, *P*<0.001) and to *M*_*s*td_ (*r*=–0.70, *P*<10^−15^, all *n*=150 sites). The non-independence of fertilization and mowing reflects the decision of the farmer to utilize the site for hay or silage yield, whereas the use as pasture represents an alternative target based on livestock.

### Sampling methods

Plants, birds and grassland arthropods were sampled yearly from 2008 to 2013, bats and forest arthropods from 2008 to 2012 and forest plants from 2009 to 2012. Cover of plant species was estimated in quadrats, arthropods were collected by standardized sweep-netting in grasslands and flight-interception traps in forests, bird abundance was determined by audio–visual counts of males, and bats by their flight activity:

*Plants*. Grassland vegetation was surveyed yearly from 2008 to 2013 from mid-May to mid-June simultaneously in all regions. The cover of all vascular plant species was estimated on 4 m × 4 m permanent plots in the grasslands^39^. In forests, plant cover was estimated in spring and late summer from 2009 to 2012 in an area of 20 m × 20 m per plot^40^. We used different plot sizes for the two types of vegetation following general recommendations for assessing vegetation for grassland and forest understorey^41^. Only understorey plants including shrub and tree seedlings were analysed; species of the tree and shrub layers are not expected to show noteworthy variation in abundance over the sampling period. Counts of plant species are conservative as they include aggregates that are counted as a single species.

*Arthropods*. Grassland arthropods were sampled yearly from 2008 to 2013 in June and August (July and September in Schorfheide-Chorin in 2009) by sweep-netting with a total of 60 double sweeps along three plot borders (total 150 m). One double sweep is defined as moving the net from the left to the right and back perpendicular to the walking direction. The sampled arthropods were preserved in 70% ethanol. We included adult individuals of the following taxa: Araneae, Hemiptera: Auchenorrhyncha (Cicadomorpha, Fulgoromorpha), Hemiptera: Heteroptera, Coleoptera and Orthoptera. For each plot, we included only years in which samples were taken in both months; for 124 grassland plots we included all 6 years, for 25 plots we included 4 years and for one plot we could only include 3 years.

Forest arthropods were sampled yearly from 2008 to 2012 over the whole vegetation period (May to October) using flight-interception traps (for general trap design, see ref. 42). These traps consist of a crossed pair of transparent plastic shields (40 cm × 60 cm) with funnels opening into sampling jars at the bottom and at the top. As sampling fluid we used a non-attractant 3% copper sulphate solution. Two traps were installed in the vegetation layer (at ∼1.5 m height) on 30 plots (12 in Hainich-Dün and 9 in Schorfheide-Chorin and 9 in Schwäbische Alb), and sampling jars were replaced with an interval of ∼5 weeks. We chose Coleoptera as the target group because of the high species and ecological diversity, and because this group is representatively assessed by flight-interception traps. All sampled adult Coleoptera species were included in the analysis and individuals were pooled per plot and year (both traps and all sampling dates).

For grassland and forest communities, a separate analysis for herbivores and remaining trophic levels (mainly predators) was performed. The assignment to trophic levels was based on the main larval and adult feeding source following Gossner *et al.*^43^ for grassland and Böhme^44^ for forest communities.

*Birds*. At each of the 300 sites we surveyed birds by standardized audio–visual point counts for 5 min per point count locality and time period^45^ and recorded all birds exhibiting territorial displays. We used 50-m-fixed radius point counts and noted all birds of each species during the 5-min interval. Each site was visited five times between March 15 and June 15 in 2008 to 2013. A minimum of 5 and a maximum of 15 sites were surveyed per day by one observer from sunrise to 11:00 am. The sequence in which sites were visited was randomized. Each song or call heard on a site was interpreted as one male territorial display behaviour. The maximum number of birds displaying per site per year (that is, the maximum number of individuals per species observed in any of the five surveys) was used as a measure of the abundance of each bird species. Aerial species were excluded from analysis, since they had been surveyed irregularly and are biased towards grasslands and beech forests (where detectability for aerial species is higher than in spruce forests).

*Bats*. We conducted standardized acoustic surveys of bats between June and September from 2008 to 2012. Acoustic recordings began immediately after local sunset and continued until 1 AM to account for the main peak of the bat activity during the night. Three to four experimental plots in different forest types were sampled each night, and each experimental plot was visited twice per year with an interval of 5 weeks^46^. Sound recordings were made in real time (sample rate: 384 kHz, 16 bit) with a Petterson-D1000x bat detector (Pettersson Electronic AG, Uppsala, Sweden) and triggered manually by listening through headphones to the output of the heterodyne system while continuously scanning the frequency range between 20 and 80 kHz. We used Avisoft SAS Lab Pro, version 5.0.24 (R. Specht, Avisoft Bioacoustics, Berlin, Germany) for sound analysis. Spectrograms were generated with a Hamming window (1,024 fast fourier transform operations, 96% overlap). We evaluated the number of bat passes as a measure for bat activity, and thus the intensity of habitat use, and identified bat species according to Jung *et al.*^46^.

### Definition of asynchrony and diversity

The correlation between the abundances *A* of species *i* and *j* across all years (*r*_*ij*_) defines their synchrony, ranging from −1 (maximum asynchrony) to +1 (perfect synchrony). Different alternatives have been proposed to summarize the average synchrony in a multispecies community. We used the recently proposed approach by Gross *et al.*^47^, which overcomes limitations of earlier synchrony metrics. In a community comprising a total of *S* species, the average synchrony *η* is defined as the mean correlation coefficient *r* between the abundances *A*_*i*_ of each species *i* versus the rest of the community (all *A*_*j*_ except *i*), hence, *η*=(1/*S*) Σ_*i*_ *r*(*A*_*i*_, Σ_*j≠i*_ *A*_*j*_) (ref. 47). In addition to *η*, we weighted species by their relative total abundances over all years (*p*_*i*_) to define the modified index


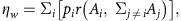


considering that the synchrony of common species has a higher influence on community stability than synchrony of rare species. Asynchrony implies negative synchrony (–*η*_w_).

Another previously used synchrony metric is *φ*=(1—*ř*_*ij*_)/*S*+*ř*_*ij*_, based on the arithmetic mean correlation coefficient *ř*_*ij*_ across all species pairs^8^,^48^. The raw mean correlation coefficient *ř*_*ij*_ is known to be biased by the variation in species richness *S*, since the minimum possible (negative) *ř*_*ij*_ is –1 for *S*=2, –0.5 for *S*=3, and asymptotically approaches zero at higher levels of *S*, whereas *φ* compensates for this effect^8^. However, this problem is also principally avoided by *η*, and hence *η*_*w*_. Both the raw *ř*_*ij*_ as well as the derived *φ* can also be weighted by the abundance of each pair of species *i* and *j*, defining their weight as *w*_*ij*_=(*p*_*i*_+*p*_*j*_)/(*S−*1). Hence, the weighted correlation coefficient would be *r*_*w*_=Σ (*w*_*ij*_ · *r*_*ij*_) and *φ*_*w*_=(1—*r*_*w*_)/*S*+*ř*_*ij*_ or alternatively *φ*_*w2*_=(1—*r*_*w*_)/*e*^*H′*^+*ř*_*ij*_, where *e*^*H′*^ is the exponential Shannon entropy also weighted by relative abundances *p*_*i*_ (ref. 49). Following Loreau and de Mazancourt^48^, Hautier *et al.*^18^ defined synchrony in a different way, based on the relationship between the population variance (*σ*_*i*_) of each species *i* and community variance (*σ*_*c*_) as *φ*_*v*_=*σ*_*c*_^2^/(Σ *σ*_*i*_)^2^, hence a definition that is not independent of community stability itself. Despite the different approaches, synchrony indices were highly correlated with each other and with *η*_*w*_ for our data (see Supplementary Fig. 1). Weighted *η*_*w*_ was highly correlated to unweighted *η* (all *r*≥0.61), to *φ*_*w*_ (*r*≥0.63) and to *r*_*w*_ (*r*≥0.68), and unweighted *η* with *φ*_*v*_ (*r*≥0.68). The conclusions taken from structural equation modelling were independent of the choice of the unweighted or weighted synchrony metric (compare Supplementary Tables 3 and 4 with Supplementary Figs 2–4).

Consistent with asynchrony, species diversity was weighted with relative total abundances (*p*_*i*_) per plot across all years based on Shannon's entropy *H′*=Σ *p*_*i*_ log(*p*_*i*_). For our analyses we implemented the exponential index, that is, the effective diversity (*e*^*H′*^) (ref. 49). The stability gain was defined as CV_sp_/CV_tot_, where CV_sp_ is the weighted mean variation coefficient of each species (weighted by *p*_*i*_) and CV_tot_ the variation of the total number of individuals in the community. All calculations and analyses were performed in R version 3.2.1 (ref. 50).

### Structural equation modelling of land-use intensity effects

We used structural equation modelling based on a maximum likelihood method to estimate the indirect effect of land-use intensity on stability via changes in asynchrony (–*η*_*w*_), diversity (*e*^*H′*^) and total abundance (mean Σ_*i*_ *A*_*i*_ per year). Path coefficients were estimated separately for forests and grasslands, and for arthropods, birds, bats and plants. We model the effects of land-use intensity as a composite variable^51^. Models were performed using the R-package ‘lavaan' version 0.5–18 (ref. 52).

Models for grasslands and forests only differed in the definition of the combined land-use intensity and its components (forests: *Iharv*, *Inonat*, *Idwcut*; grasslands: fertilization, mowing and grazing, see above). Following Grace and Bollen^51^, the combined land-use intensity could be defined either as a latent variable or as a composite variable. We decided to use a composite variable, assuming that the components (after combining the correlated components fertilization and mowing, see above) behave independently from each other rather than being interchangeable manifestations of land-use intensity. Hence, we followed the recommendation by Grace and Bollen^51^ that ‘a lack of correlation among indicators (of a construct) would contraindicate the prospect that a block should be of the L→M form (that is, a latent variable in contrast to a composite)'. In lavaan^52^, correlations between the components of composite variables are not considered. As a composite variable is a representation of the collective effects of its components, it is assumed that it has no further error variance. Therefore, the error variance was fixed to zero in the model, and the scale of the measurement for the composite was defined by setting the path coefficient from *Iharv* or grazing, respectively, to the composite to 1. The path coefficients and their associated *P* values were estimated with the maximum likelihood method, and the overall model fit was estimated from a *χ*^2^-test between the modelled and observed covariance matrix. It is important to notice that this *χ*^2^-test shows significant *P* values (*P*<0.05) if there is significant discrepancy between the model and the data, which is undesirable. As we are more interested in the relative effects of the land-use components on stability through changes in asynchrony, diversity and abundance rather than their absolute effects, we only consider standardized path coefficients. Before the calculation of the s.e.m.'s, all variables (except the land-use components) were visually inspected for normality and transformed where necessary. When composite variables with more than one outward-pointing pathway (here diversity, abundance and asynchrony) are used in a model, it is assumed that the effects of the indicator variables on the measured variables are proportional to each other. If this assumption is not met, the parameter values are likely biased^51^. This is often reflected by very high error variances associated with the measured variables. To test whether we correctly assumed proportional effects, we calculated an additional basic model that did not include any composite variables but direct pathways between the three indicator variables (the land-use components) and the three measured variables. Smaller error variances in this basic model compared with the original model indicate that the effects of the land-use components are not proportional between the three measured variables. As we fixed the path coefficient between *Iharv* or grazing and the composite in the original model, it was not possible to estimate its statistical significance in the original model. We evaluated the statistical significance of these path coefficients using the basic model as well.

The original structural equation models showed nonsignificant overall *P* values for most of the taxa, indicating that the models represent the covariance structure in the data adequately. In cases for which the overall *P* value showed a significant difference between the model and the data, we first calculated a reduced model in which three composite variables were included (one composite for each of the three measured variables diversity, abundance and asynchrony). If the overall *P* value was also significant with the reduced model, we used the basic model for this group. We found very high error variances (>0.75) for asynchrony, diversity and abundance in all models, indicating that effects of the land-use components are not proportional between those three variables. Basic models without a composite variable, however, reduced the error variances only for some groups (grassland arthropods as well as forest birds). Within the basic model for grassland arthropods, none of the components significantly affected diversity; however, mowing and fertilization showed significant effects on asynchrony and abundance. Basic models for forest birds showed no significant effect on diversity, but did show significant effects of *Iharv* and *Inonat* on abundance as well as *Iharv* and *Idwcut* on asynchrony.

## Additional information

**How to cite this article:** Blüthgen, N. *et al.* Land use imperils plant and animal community stability through changes in asynchrony rather than diversity. *Nat. Commun.* 7:10697 doi: 10.1038/ncomms10697 (2016).

## Supplementary Material

Supplementary Information Supplementary Figures 1–6 and Supplementary Tables 1–5
