# Supplementary material for: Land use imperils plant and animal community stability through changes in asynchrony rather than diversity
Source: Nat Commun. 2016 Feb 12;7:10697. doi: 10.1038/ncomms10697 (PMC4754335; doi:10.1038/ncomms10697)
Supplement: Supplementary Information — Supplementary Figures 1–6 and Supplementary Tables 1–5 [file ncomms10697-s1.pdf]

Supplementary Figures

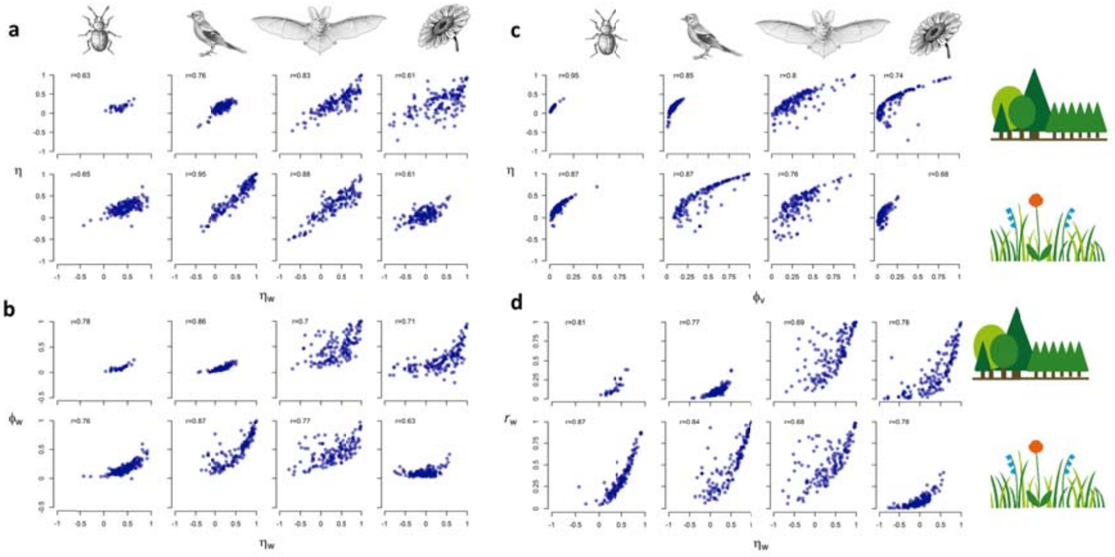

Supplementary Figure 1| Correlations between alternative synchrony indices. Comparison of alternative synchrony indices for communities of arthropods, birds, bats and plants in forests and grasslands. Given are the correlation coefficients (*r*). (a) Comparison between weighted and unweighted η. (b) Comparison between weighted η and weighted ϕ. (c) Comparison between unweighted η and unweighted ϕ. (d) Comparison between weighted η and weighted r_w. The different indices are described in Material and Methods.

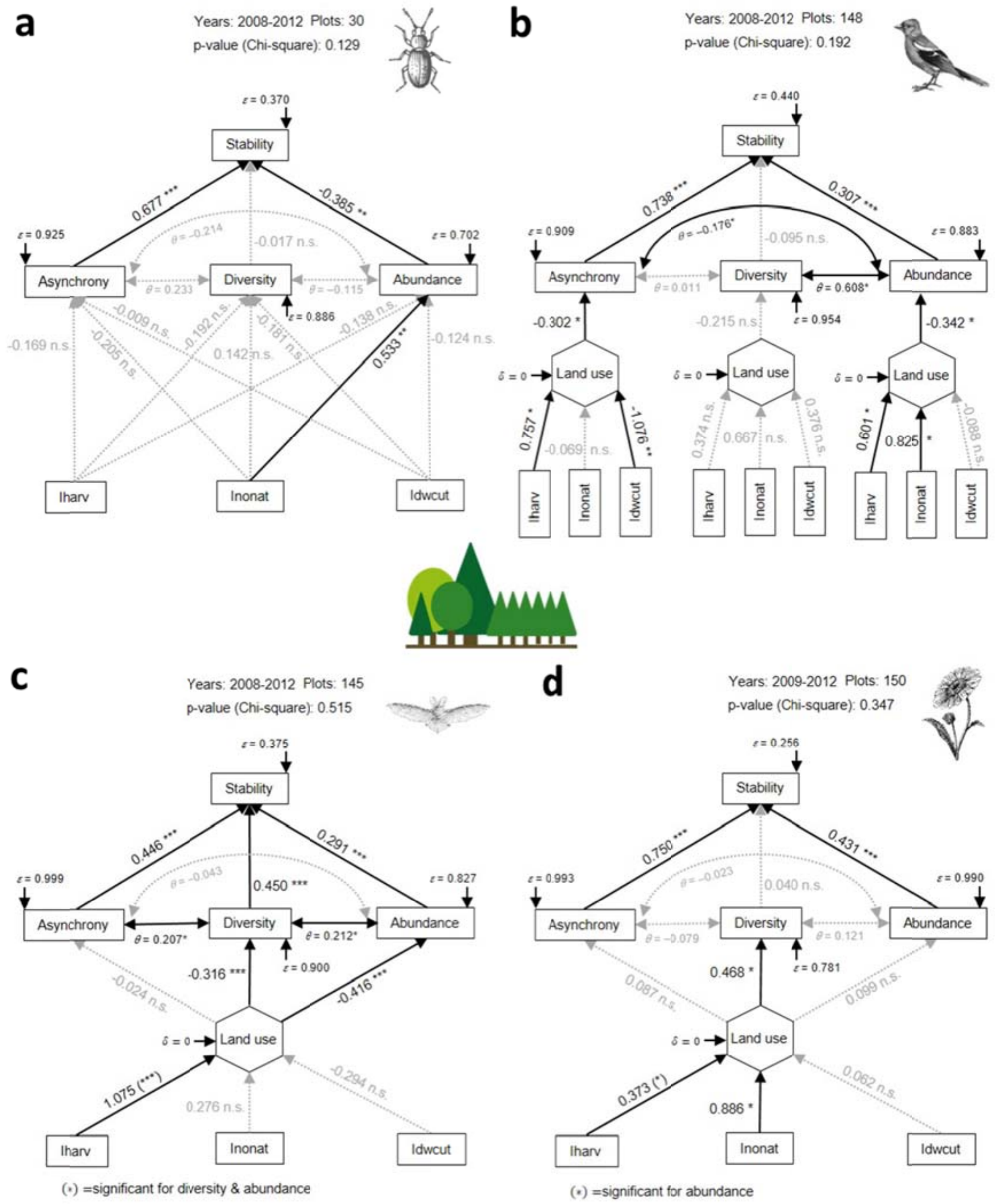

Supplementary Figure 2| Structural equation models for forests. Estimated path coefficients and significance of single pathways within structural equation models for arthropods (a), birds (b), bats (c) and plants (d). Standardized linear coefficients are shown next to the arrows together with their *p*-value (n.s. not significant; * $p < 0.05$; ** $p < 0.01$; *** $p < 0.001$). *Iharv*: proportion of harvested tree volume, *Inonat*: proportion of tree species that are not part of the natural forest community, *Idwcut*: the proportion of dead wood showing signs of saw cuts.

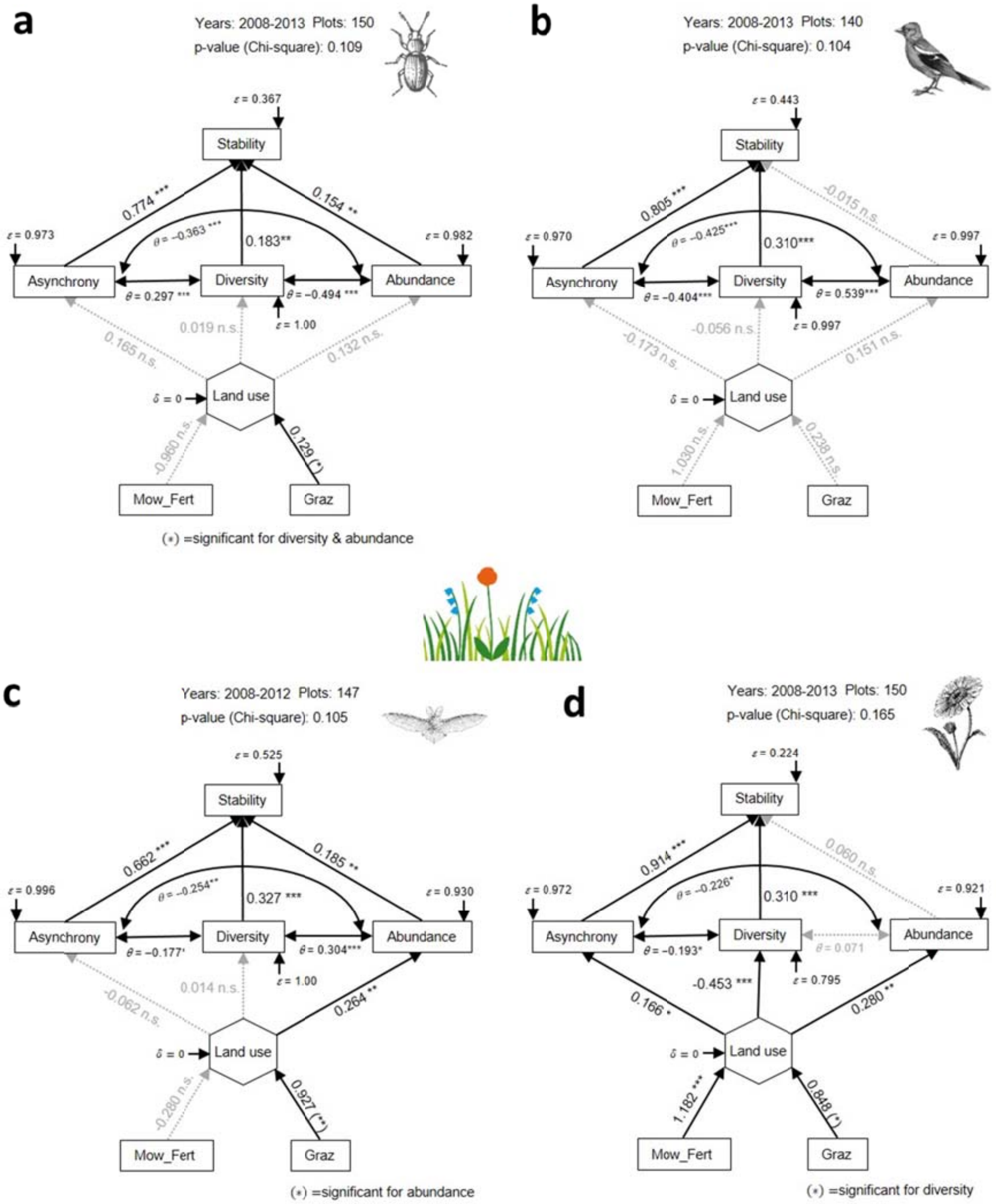

Supplementary Figure 3| Structural equation models for grasslands. Estimated path coefficients and significance of single pathways within structural equation models for arthropods (a), birds (b), bats (c) and plants (d). Standardized linear coefficients are shown besides the arrows together with their *p*-value (n.s. not significant; * $p < 0.05$; ** $p < 0.01$; *** $p < 0.001$). Mowing and fertilization (Mow_Fert) were averaged after standardization because they were correlated (see Supplementary Methods and Supplementary Data). Graz: Grazing.

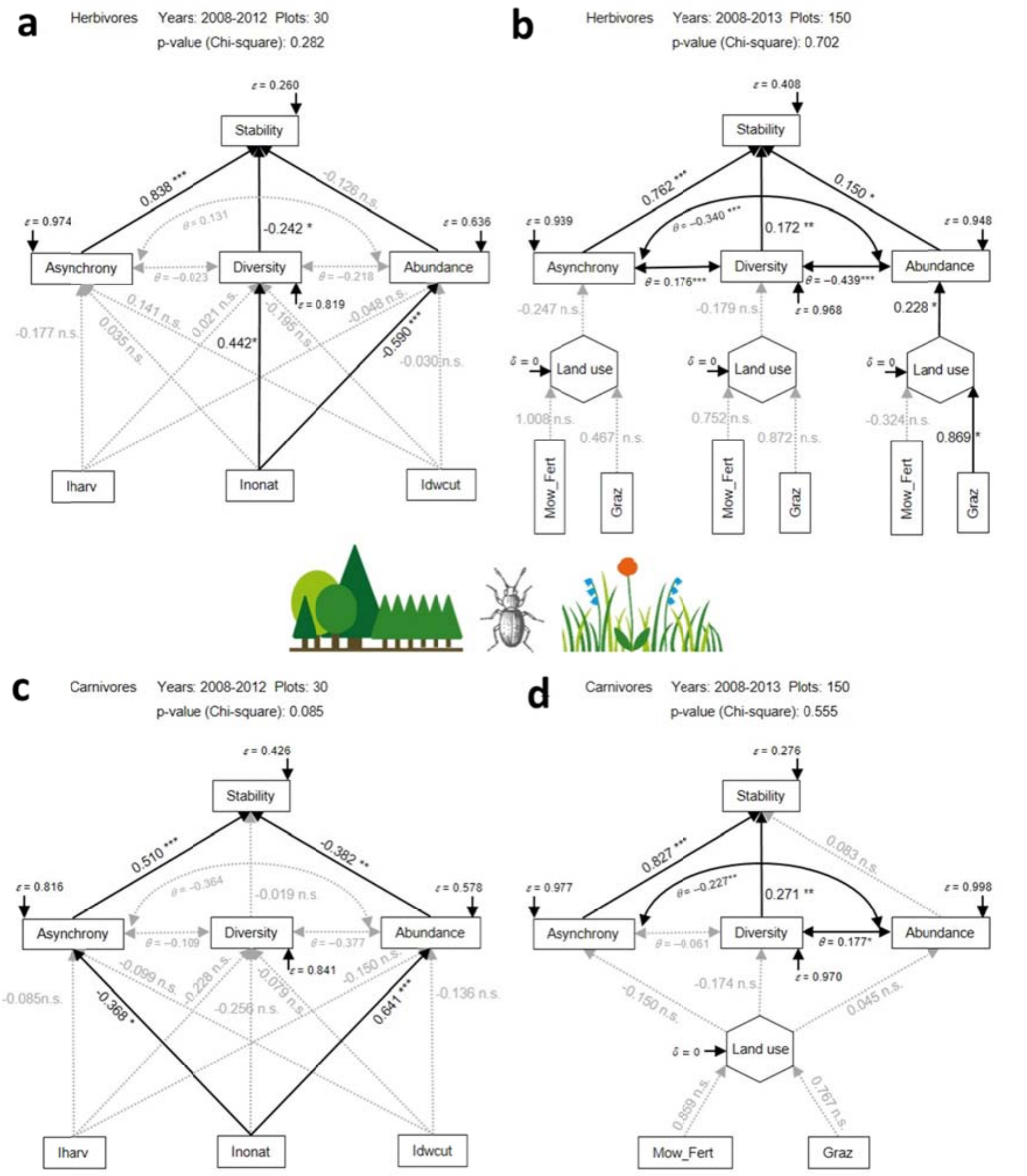

Supplementary Figure 4| Structural equation models for functional groups in arthropods. Estimated path coefficients and significance of single pathways within structural equation models for functional groups within grassland and forest arthropods. Herbivorous arthropods are shown in the top (a-b) and carnivorous arthropods are shown in the bottom (c-d). Panels on the left show models for forest, panels on the right models for grassland. Standardized linear coefficients are shown together with their p-value (n.s. not significant; * $p < 0.05$; ** $p < 0.01$; *** $p < 0.001$). *Iharv*: proportion of harvested tree volume, *Inonat*: proportion of tree species that are not part of the natural forest community, *Idwcut*: the proportion of dead wood showing signs of saw cuts. Mowing and fertilization (Mow_Fert) were averaged after standardization because they were correlated (see Supplementary Methods and Supplementary Data). Graz: Grazing.

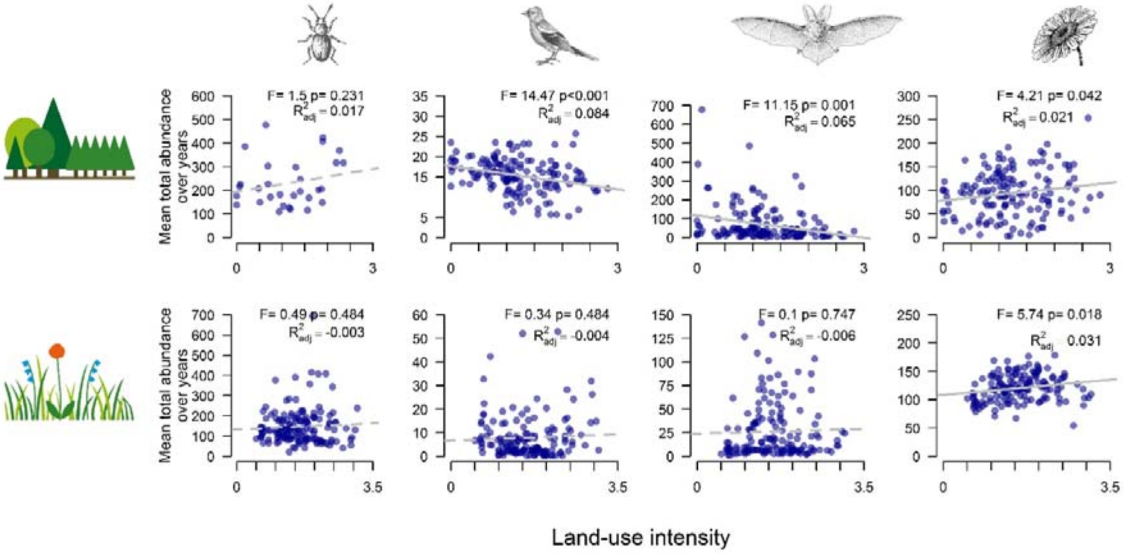

Supplementary Figure 5| Effect of land-use intensity on average abundance within forests and grasslands. Mean abundance (calculated across years) against the combined land-use intensity in forests (top row) and grasslands (bottom row) is shown for each of the four taxa. Significance values are derived from linear models.

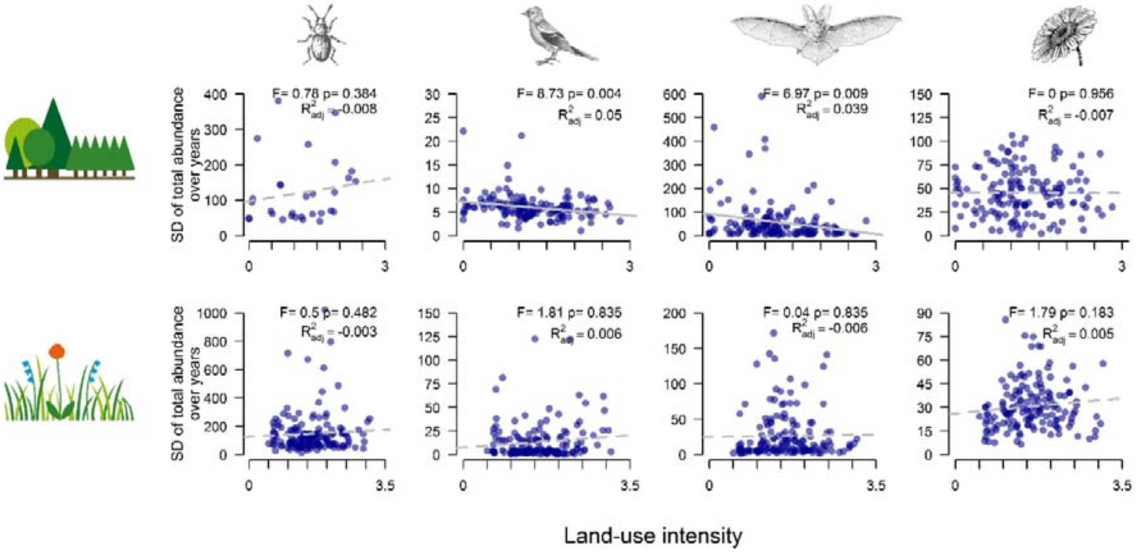

Supplementary Figure 6| Effect of land-use intensity on the variability in abundance within forests and grasslands. Variability in abundance (calculated as the standard deviation, sd across years) against the combined land-use intensity in forests (top row) and grasslands (bottom row) is shown for each of the four taxa. Significance values are derived from linear models.

Supplementary Tables

Supplementary Table 1

Analysis of variance in community and population variability across taxa and habitats. Summary statistics from analyses of variance (ANOVA) testing the effect of habitat types (forest and grassland) and taxa (arthropods, birds, bats and plants) on community variability (CV_{tot}), average species variability (CV_{sp}) and the stability gain, i.e. the reduction in community variability in comparison to species variability ($CV_{\mathrm{sp}}/CV_{\mathrm{tot}}$). Due to significant effects and habitat × taxon interactions, differences between habitats and taxa are provided in Supplementary Table 2.

	Df	SumSq	MeanSq	F value	*p*-value
CV_{tot}					
Taxon	3	95.5	31.9	259.8	<0.001
Habitat	1	18.1	18.1	147.3	<0.001
Taxon × Habitat	3	45.4	15.1	123.6	<0.001
Residuals	1,056	129.4	0.1		
CV_{sp}					
Taxon	3	40.5	13.5	173.9	<0.001
Habitat	1	15.6	15.6	201.7	<0.001
Taxon × Habitat	3	78.5	26.2	338.5	<0.001
Residuals	1,056	81.7	0.1		
CV_{sp}/CV_{tot}					
Taxon	3	52.2	17.4	328.1	<0.001
Habitat	1	0.9	0.9	17.1	<0.001
Taxon × Habitat	3	1.08	1.4	25.6	<0.001
Residuals	1,056	56.0	0.1		

Supplementary Table 2

Comparisons of variability, asynchrony and diversity across taxa and their variation (standard deviation sd) across *N* sites. Mean and standard deviation for community variability (CV_{tot}), weighted mean species variability (CV_{sp}), stability gain (CV_{sp}/CV_{tot}), weighted asynchrony (η_w), exponential Shannon diversity ($e^{H'}$), the mean total community abundance $\mu(A_{tot})$ and its variability (standard deviation $\sigma(A_{tot})$). Asterisks show significance level for *t*-tests (Welch corrected) for the first four parameters between forests and grasslands (n.s. not significant; * $p < 0.05$; ** $p < 0.01$; *** $p < 0.001$). *N* = number of plots sampled per taxon. Note that methods differ in both habitats, with likely consequences on diversity; forest plants did not include trees and shrubs above 0.5 m height, which would have led to slightly lower variability levels. Also note that $\sigma(A_{tot})$ is more variable across sites than the $\mu(A_{tot})$ for each of the systems (higher CV).

	Forest				Grassland			
	Arthropods	Birds	Bats	Plants	Arthropods	Birds	Bats	Plants
N	30	148	146	148	150	135	145	150
CV_{sp}/CV_{tot}								
mean	2.91	3.60	1.48	2.71	1.98	1.59	1.53	4.37
sd	0.82	1.17	0.46	2.65	0.66	0.57	0.48	2.08
CV_{tot}					***	***	n.s.	***
mean	0.48	0.40	1.00	0.59	0.94	1.48	1.02	0.25
sd	0.17	0.13	0.42	0.40	0.35	0.51	0.37	0.11
CV_{sp}					***	***	n.s.	***
mean	1.29	1.31	1.33	1.07	1.68	2.09	1.44	0.91
sd	0.16	0.17	0.36	0.34	0.24	0.30	0.32	0.16
Asynchrony					***	***	**	***
mean	-0.34	-0.15	-0.42	-0.36	-0.46	-0.49	-0.33	0.04
sd	0.14	0.15	0.37	0.45	0.23	0.37	0.41	0.26
Diversity					***	**	**	***
mean	55.75	20.72	3.92	15.94	22.90	8.87	4.07	14.54
sd	19.48	3.34	2.11	9.09	9.78	5.90	1.62	5.69
$\mu(A_{tot})$								
mean	230.8	15.3	68.9	93.6	147	7.9	26.3	120.6
sd	101.7	4.2	95.3	48.1	89	9.1	29.5	23.5
CV	0.44	0.27	1.38	0.51	0.61	1.15	1.12	0.19
$\sigma(A_{tot})$								
mean	121	6	57.2	45.0	148.1	13.4	26.5	30.7
sd	91.1	2.7	84.4	24.8	142.9	19.6	33.1	14.8
CV	0.75	0.44	1.48	0.55	0.96	1.46	1.25	0.48

Supplementary Table 3

Results of structural equation models including unweighted asynchrony for forests. Estimated path coefficients and significance of single pathways within structural equation models for forests which included the unweighted asynchrony index (–*η*). The combination of paths given depends on the simplest model with a non-significant difference between model and data (overall p-value from maximum likelihood models). Column under each taxon give the standardized estimate and their p-value (n.s. not significant; * $p < 0.05$; ** $p < 0.01$; *** $p < 0.001$). † = significant effect for diversity. ‡ =significant effect for abundance.

**Regressions:**	Plants		Bats		Arthropods		Birds		Carnivore	
p-value (Chi²)	0.27		0.13		0.17		0.17		0.06	
Iharv -> Landuse	0.35	‡	1.07	† ,‡	0.28	n.s.	0.64	‡	0.29	n.s.
Inonant -> Landuse	0.86	n.s.	0.30	n.s.	-0.92	n.s.	0.83	*	-0.96	n.s.
Idwcut -> Landuse	0.13	n.s.	-0.29	n.s.	0.39	n.s.	-0.16	n.s.	0.26	n.s.
Landuse -> Diversity	0.47	*	-0.31	***	-0.26	n.s.	-0.19	n.s.	0.12	n.s.
Landuse -> Abundance	0.10	n.s.	-0.42	***	-0.54	n.s.	-0.34	*	-0.65	n.s.
Landuse -> Asynchrony	-0.01	n.s.	0.08	n.s.	0.20	n.s.	0.03	n.s.	0.15	n.s.
Diversity -> Stability	0.12	*	0.49	***	0.29	*	0.16	**	0.16	n.s.
Abundance -> Stability	0.25	***	0.25	***	-0.53	***	0.23	***	-0.51	***
Asynchrony -> Stability	0.63	***	-0.36	***	0.50	***	0.84	***	0.47	**
**Covariances:**										
Diversity ~ Abundance	0.12	n.s.	0.21	*	-0.12	n.s.	0.61	***	-0.38	n.s.
Diversity ~ Asynchrony	-0.13	n.s.	-0.16	n.s.	-0.24	n.s.	-0.22	**	-0.43	*
Abundance ~ Asynchrony	0.26	**	-0.02	n.s.	0.12	n.s.	-0.17	*	0.03	n.s.
**R-square:**										
Diversity	0.22		0.10		0.07		0.04		0.01	
Abundance	0.01		0.17		0.29		0.12		0.42	
Asynchrony	0.00		0.01		0.04		0.00		0.02	
Stability	0.55		0.58		0.52		0.71		0.54	

	Herbivore	
p-value (Chi²)	0.45	
Iharv -> Diversity	0.02	n.s.
Inonat -> Diversity	0.44	*
Idwcut -> Diversity	-0.20	n.s.
Iharv -> Abundance	-0.05	n.s.
Inonat -> Abundance	-0.59	***
Idwcut -> Abundance	-0.03	n.s.
Iharv -> Asynchrony	0.22	n.s.
Inonant -> Asynchrony	0.04	n.s.
Idwcut -> Asynchrony	-0.10	n.s.
Diversity -> Stability	-0.26	*
Abundance -> Stability	-0.28	*
Asynchrony -> Stability	-0.75	***
**Covariances:**		
Diversity ~ Abundance	-0.22	n.s.
Diversity ~ Asynchrony	0.05	n.s.
Abundance ~ Asynchrony	-0.31	n.s.
**R-square:**		
Diversity	0.18	
Abundance	0.36	
Asynchrony	0.03	
Stability	0.57	

Supplementary Table 4

Results of structural equation models including unweighted asynchrony for grasslands. Estimated path coefficients and significance of single pathways within structural equation models which included the unweighted asynchrony index (–*η*). The combination of paths given depends on the simplest model with a non-significant difference between model and data (overall p-value from maximum likelihood models). Column under each taxon give the standardized estimate and their *p*-value (n.s. not significant; * $p < 0.05$; ** $p < 0.01$; *** $p < 0.001$). MF: average of mowing and fertilization after standardization. † = significant effect for diversity. ‡ = significant effect for abundance.

**Regressions:**	Plants		Arthropods		Birds		Bats		Carnivore			Herbivore	
p-value (Chi²)	0.85		0.07		0.23		0.38		0.37			0.10	
Grazing -> Landuse	0.85	†‡	1.00	†,‡	0.15	n.s.	0.99	‡	0.82	n.s.	Grazing -> Diversity	-0.16	n.s.
MF -> Landuse	1.18	***	0.00	n.s.	1.03	n.s.	-0.04	n.s.	0.81	*	MF -> Diversity	-0.14	n.s.
Landuse -> Diversity	-0.45	***	-0.14	n.s.	-0.06	n.s.	0.03	n.s.	-0.17	n.s.	Grazing -> Abundance	0.20	*
Landuse -> Abundance	0.28	**	0.22	**	0.16	n.s.	0.26	**	0.04	n.s.	MF -> Abundance	-0.07	n.s.
Landuse -> Asynchrony	0.19	*	-0.06	n.s.	-0.21	n.s.	0.06	n.s.	-0.15	n.s.	Grazing -> Asynchrony	-0.02	n.s.
Diversity -> Stability	0.15	*	0.36	***	0.44	***	0.38	***	0.38	***	MF -> Asynchrony	-0.14	n.s.
Abundance -> Stability	-0.11	n.s.	-0.02	n.s.	-0.07	n.s.	0.14	*	-0.05	n.s.	Diversity -> Stability	0.33	***
Asynchrony -> Stability	0.56	***	0.60	***	0.86	***	0.62	***	0.82	***	Abundance -> Stability	-0.05	n.s.
											Asynchrony -> Stability	0.66	***
**Covariances:**											**Covariances:**		
Diversity ~ Abundance	0.07	n.s.	-0.47	***	0.54	***	0.30	**	0.18	*	Diversity ~ Abundance	-0.44	***
Diversity ~ Asynchrony	-0.06	n.s.	-0.06	n.s.	-0.50	***	-0.24	**	-0.17	*	Diversity ~ Asynchrony	-0.15	n.s.
Abundance ~ Asynchrony	-0.04	n.s.	0.02	n.s.	-0.41	***	-0.25	**	-0.09	n.s.	Abundance ~ Asynchrony	0.03	n.s.
**R-square:**											**R-square:**		
Diversity	0.21		0.02		0.00		0.00		0.03		Diversity	0.03	
Abundance	0.08		0.05		0.02		0.07		0.00		Abundance	0.05	
Asynchrony	0.04		0.00		0.05		0.00		0.02		Asynchrony	0.02	
Stability	0.33		0.47		0.60		0.43		0.73		Stability	0.50	

Supplementary Table 5

Contingency tables for mowing, fertilization and grazing in 150 grasslands; number of sites shown for each category, covering the years 2006 – 2012 (e.g. 38 sites were never mown during the seven years). Combinations that are more common than expected by chance are highlighted in bold (expected = row total × column total / 150).

(a) Mowing *versus* fertilization

	Mown	Not mown	Total
Fertilized	**69**	4	73
Unfertilized	43	**34**	77
Total	112	38	150

(b) Grazing *versus* fertilization

	Grazed	Ungrazed	Total
Fertilized	53	**20**	73
Unfertilized	**66**	11	77
Total	119	31	150

(c) Grazing *versus* mowing

	Grazed	Ungrazed	Total
Mown	81	**31**	112
Not mown	**38**	0	38
Total	119	31	150
